# Time-Resolved Studies of Ytterbium Distribution at Interfacial Surfaces of Ferritin-like Dps Protein Demonstrate Metal Uptake and Storage Pathways

**DOI:** 10.3390/biomedicines9080914

**Published:** 2021-07-29

**Authors:** Kornelius Zeth, Gabriela Pretre, Mitsuhiro Okuda

**Affiliations:** 1 Department of Science and Environment, Roskilde University, Universitetsvej 1, 4000 Roskilde, Denmark; 2 CIC nanoGUNE, 20018 Donostia-San Sebastian, Basque, Spain; gabrielapretre@gmail.com; 3 IKERBASQUE, Basque Foundation for Science, 48011 Bilbao, Basque, Spain

**Keywords:** Dps, ferritin, biomineralization, bio-imaging carrier, medical therapy carrier

## Abstract

Cage-shaped protein (CSP) complexes are frequently used in bionanotechnology, and they have a variety of different architectures and sizes. The smallest cage-shaped protein, Dps (DNA binding protein from starved cells), can naturally form iron oxide biominerals in a multistep process of ion attraction, translocation, oxidation, and nucleation. The structural basis of this biomineralization mechanism is still unclear. The aim of this paper is to further develop understanding of this topic. Time-resolved metal translocation of Yb^3+^ ions has been investigated on Dps surfaces using X-ray crystallography. The results reveal that the soak time of protein crystals with Yb^3+^ ions strongly affects metal positions during metal translocation, in particular, around and inside the ion translocation pore. We have trapped a dynamic state with ongoing translocation events and compared this to a static state, which is reached when the cavity of Dps is entirely filled by metal ions and translocation is therefore blocked. By comparison with La^3+^ and Co^2+^ datasets, the time-dependence together with the coordination sphere chemistry primarily determine metal−protein interactions. Our data can allow structure-based protein engineering to generate CSPs for the production of tailored nanoparticles.

## 1. Introduction

Cage-shaped proteins (CSPs) of various sizes are used as molecular carriers for organic molecules in, e.g., anti-cancer therapy and imaging [1,2]. The formation of defined metal nanoparticles for nanobiotechnology applications used in memory storage and hydrothermal processes are other important fields for CSP applications [3]. Two independent surface areas in CSPs may be modified to achieve new functionalities: the outer surface in order to make CSPs target specific, and the inner surface to alter, e.g., mineralization kinetics and nucleation. More specifically, the outer surface of CSPs can be functionalized, e.g., with antibodies, peptides, or organic molecules to generate cages specifically targeting receptors [4,5]. Many CSPs are known to be involved in iron-oxide assembly in cells and can be broadly used for the formation of metal-oxide/sulfide nanoparticles in the range of 5 to 50 nm.

**Figure 1:**
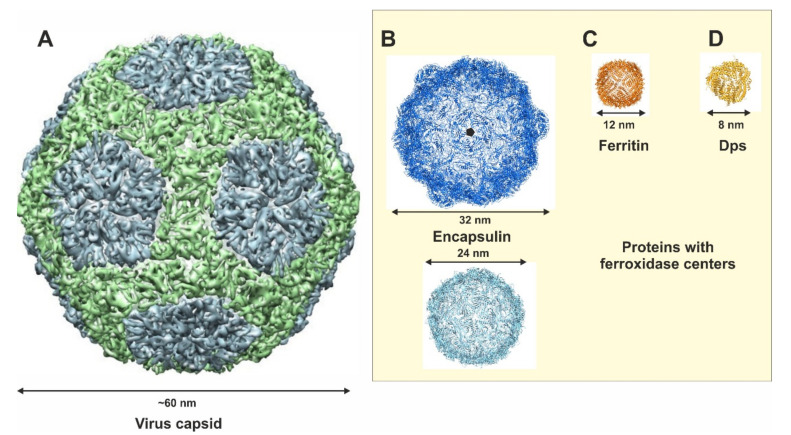
Structures of CSPs used as biocompartments represented as surface or ribbon models. (**A**) Bacteriophage P22 viral capsids shown in surface representation. (**B**) Encapsulin from *Myxococcus xanthus* (upper) and from *Themotoga maritima* (lower) shown in cartoon representation with the individual dimensions given. (**C**) Ferritin consisting of 24 identical subunits and an outer diameter of 12 nm. (**D**) Dps protein complex consisting of 12 identical subunits and an outer diameter of 8 nm.

Viruses and phages comprise highly symmetric protein cages which naturally enclose DNA or RNA [6]. These, being the largest CSPs of ~65 nm diameter (Figure 1A), are often used for biomedical applications including vehicles for targeted anti-cancer therapy and templated nanoparticle synthesis [7,8]. Another smaller biomolecular capsid is encapsulin, found in many prokaryotic organisms. For all CSPs naturally forming iron-oxide, including encapsulin, ferritin and Dps, the iron in the cavity is stored as an iron (III) oxide nanoparticle through a process termed biomineralization. Thereby, the nanoparticle diameter is strictly regulated by the cavity size to yield iron-oxide nanoparticles of a very distinct size. Encapsulin naturally carries smaller bacterial proteins and protein cages (e.g., ferritin) to maintain oxidation activity (Figure 1B) [9]. This complex is currently the largest known bacterial iron-oxide storage architecture with a spherical form of 25–42 nm in diameter [9]. Apo-encapsulin has been used for the formation of magnetic nanoparticles. In addition, medicines have also been enclosed, demonstrating the potential of this complex as a bio-imaging and medical therapy carrier [7,8].

Biomineralization in proteins of the ferritin superfamily (bacterioferritin, ferritin and Dps) is a multistep mechanism initiated by the electrostatic interaction of the positively charged iron atoms with residues surrounding the uptake pores of ferritin and Dps [3,10]. Negatively charged (mostly glutamate) and hydrophilic residues are observed along the pore, which is of threefold symmetry, and these residues guide iron toward the cavity before selectivity filters further narrow the pore diameter down to about 3 Å and likely strip off the water coordination sphere at this point [3,10]. The pore gate allows only metal ions to enter and to proceed inside the cavity along negatively charged residues toward the ferroxidase centers. These centers bind iron or other metal ions and allow for oxidation. The oxidized iron is then submitted to nucleation centers which are the anchor points of the final growing biomineral, thus completing the process of biomineralization in these proteins.

Ferritin is the best-studied CSP regarding biochemistry and structural biology, but also in bionanotechnology. The protein is central in pro- and eukaryotic cells. The protein has a natural function of attracting and storing iron as iron oxide, but also to release the mineral when iron is scarce in order to maintain vital functions [3,10]. Ferritin consists of 24 identical subunits and forms spherical shaped particles with a 12 nm outer cage diameter surrounding an inner spherical cavity of 8 nm (Figure 1C) [3,10]. The protein cage contains two-, three- and four-fold molecular symmetry axes, which are important for the assembly of functional units such as the translocation pores. Ferritin has also been used for various applications in nanobiotechonology. Recombinant apoferritin from plants and eukaryotes can be used as a development platform for drug and imaging carriers through modification at the interfacial region on the ferritin surface [11]. The outer surface of ferritin has been functionalized with various groups. Peptides such as the RGD peptide motif were attached to specifically recognize melanoma cells and to release the cargo of small anti-cancer molecules [12,13]. The ferritin cavity can also be loaded with drugs such as cisplatin [14] and doxorubicin [15] as anti-cancer drugs. For imaging, agents such as the Gd chelate complex for magnetic resonance imaging [16] and cy5.5 (cyanine-5.5) for fluorescence cell imaging were applied [11], respectively. In addition, the cavity can be utilized as a template to fabricate inorganic nanoparticles made of rare earth elements instead of iron oxide with luminescent or special magnetic properties [17].

The smallest iron-oxide binding CSP is Dps (DNA binding protein starved cell) which belongs to the ferritin superfamily but was reported to also maintain an important function in DNA condensation and protection under starvation conditions (Figure 1D) [3,18]. Dps proteins suppress oxidation stress from H_2_O_2_ or hydroxyl radicals after rapidly removing iron from the cytoplasm and the formation of iron oxide in their cavity [18]. Dps contains 12 identical subunits forming a 9 nm outer diameter and a cavity size of 4.5 nm (Figure 1D) [19]. The protein contains four threefold and six twofold symmetry axes and the translocation pores for iron uptake are organized along the three-fold axis. While the presence of translocation pore 1 (termed T1) has been identified in various bacterial Dps proteins (e.g., Dps from *Microbacterium arborescence*) due to co-crystallization with metal ions, the function of translocation pore 2 (termed T2) is still questionable [19]. It has been shown that Dps can also be used as a drug/imaging carrier platform [20]. To date, *Li*Dps (*Listeria innocua* Dps) has been employed to fabricate nanoparticles such as Fe_2_O_3_-r-Fe_3_O_4_ [21], CdS [22], CdSe [23] and CuS [24], however, biomineralization in *Li*Dps has not been well investigated and the underlying mechanism occurring inside the cavity has still not been unraveled. Therefore, any knowledge acquired about the biomineralization process in *Li*Dps will lead to better tuning of the properties of Dps for nanotechnological applications, e.g., the improvement of luminescence imaging and/or as a magnetic therapy by the biomineralization of novel nanoparticles in the Dps cavity [20,21].

Previously, we observed divalent Cobalt (Co^2+^) and Zinc (Zn^2+^) ions, and trivalent Lanthanum (La^3+^) ion positions and the plasticity of amino acids at interfacial parts on *Listeria innocua* Dps (*Li*Dps) [18]. This demonstrated the pathway of metal translocation from uptake pores toward the ferroxidase center (FOC) [19]. Here, to further establish the plasticity properties and biomineralization on the *Li*Dps interfacial surface, we demonstrate that the amino acid plasticity and the positions of trivalent Ytterbium (Yb^3+^) as being dependent on the soak time (30 and 120 min incubation), which allowed us to form time-resolved snap shots of dynamic intermediate and static structures (final state). The ninefold coordination sphere of Yb^3+^ (ion radius 1.00 Å) is almost identical to La^3+^ (ion radius 1.17 Å), which has the largest ion radius among lanthanides. In comparison to lanthanides, Co^2+^ has a sixfold coordination sphere that significantly changes the interactions with surface residues. Study of the available Yb^3+^ data may indicate differences in charge effect with Co^2+^ and of the coordination affinity with trivalent La^3+^. Surprisingly, we observed a difference in the number of Yb^3+^ positions, in particular, in the main uptake pore which is dependent on the soak time. In addition, trivalent Yb^3+^ ions show a difference from divalent Co^2+^ ions for the positions and plasticity of amino acids on *Li*Dps, similar to the La^3+^/Co^2+^ comparison. Therefore, the change in coordination number requires a strong plasticity of amino acids on *Li*Dps.

## 2. Materials and Methods

### 2.1. Expression, Purification, and Crystallization of Apo-LiDps

*Li*Dps was expressed, purified, and crystallized as previously described [19,22]. The crystallization method is briefly described below. The protein of 15−20 mg/mL in 5 mM HEPES (pH 7.5) was crystallized using the hanging drop method in VDXm plates (Hampton Research, Aliso Viejo, CA, USA). Drops were prepared by mixing equal volume 0.5–2.0 μL of protein with 0.5–2.0 μL of a reservoir solution and equilibrated against 500 μL of a reservoir solution containing 100 mM HEPES (pH 7.5) and 12.5–20.0% polyethylene glycol (PEG) 1500. Plates were incubated at 21 °C and the first crystals were obtained after 24 h. Two types of crystals (space groups of the crystals: P41212 and P212121) were formed under essentially the same conditions, one of which was space group P41212 with six monomers in the asymmetric unit (AU).

### 2.2. Metal Soaking and LiDps Structure Determination

After crystallization of *Li*Dps without metal ions, the crystals were picked from the crystallization solution and soaked for 30 or 120 min in a reservoir solution containing 10 mM YbCl3. Native and metal-soaked crystals were briefly immersed in a reservoir solution containing 30% PEG400 and flash-frozen in liquid nitrogen. Data was collected at the ALBA synchrotron light source facility (Barcelona, Spain; beamline BL13-XALOC), and diffraction images were recorded on a PILATUS 6M detector (Dectris, Baden, Switzerland) [25]. High-redundancy data sets were collected at 100 K and processed using the XDS/XSCALE or MOSFLM program packages [26,27]. Longer wavelengths resonant with K-edge absorption features of Yb^3+^ would have generated clearer anomalous maps; however, we used 0.98 Å, which can also be used to calculate anomalous maps for the detection of metal-binding sites. The structures were determined by molecular replacement using the *Listeria innocua* Dps structure [Protein Data Bank (PDB) entry 1QGH] as a search model [28]. Metal atoms were placed according to strong densities in anomalous maps as a first selection criterion (normally only >4σ peaks were taken into consideration), and isomorphous differences in the maps were calculated using Dps without metal ions (apo-Dps) and metal-soaked crystals. The individual occupancies of metal atoms were refined and validated by visual inspection of difference maps and comparison of metal B-factors with their environments. The structures were modeled using COOT software [28], REFMAC [29,30], and the final stereochemistry was analyzed using MolProbity (http://molprobity.biochem.duke.edu) [31]. All of the figures were prepared using PYMOL (http://www.pymol.org) [32]. Crystal data is found in supplemental materials (Supplementary materials Table S1).

## 3. Results

### 3.1. The Architectures of T1 and T2 Pores of LiDps

**Figure 2:**
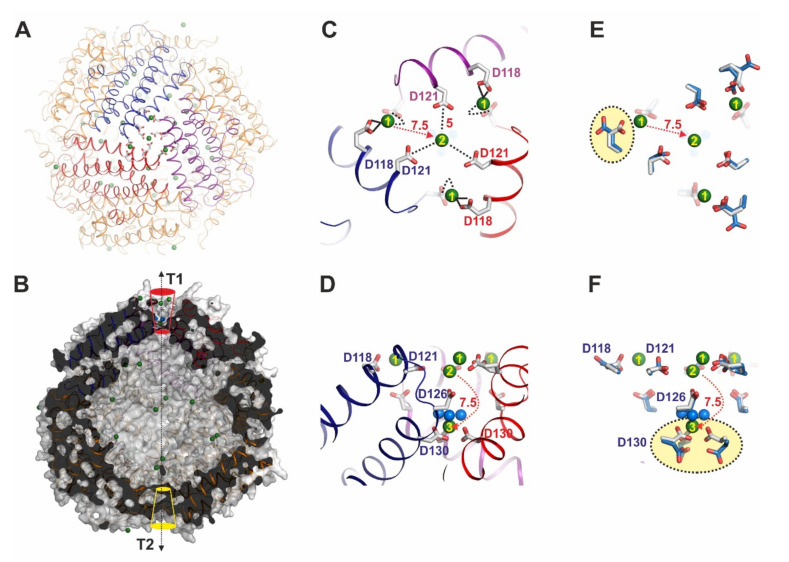
Architectures and uptake mechanism of Yb^3+^ ions through the T1 uptake pore of *Li*Dps with Yb^3+^ incubated for 30 min with *Li*Dps crystals (Dps_Yb_30). (**A**) Architecture of the T1 pore viewed from the top of Dps_Yb_30 shown in cartoon representation. (**B**) The surface representation of Dps_Yb_30 is shown; the subunits are marked in cartoon representation, with the arrangement of T1 (in red color) and T2 (in yellow color) pores located at opposite sides of the protein cage. (**C**) Architecture of the T1 pore viewed from the top. Yb^3+^ ions are shown as light green spheres with positions termed 1–3 and distances of 7.5 Å between positions 1 and 2, and 7.5 Å between positions 2 and 3, respectively. The residues of one subunit along the 3-fold axis are represented by sticks and marked according to the amino acid sequence. Dotted lines indicate interaction of Yb^3+^ with side chains. (**D**) Architecture of the T1 pore viewed from the side. (**E**) The residues in Figure 2C of one subunit along the 3-fold axis are represented by sticks and marked according to the sequence. The side chain Glu118 and Asp130 are colored yellow and are significant dynamics. (**F**) The residues in Figure 2D of one subunit along the 3-fold axis are represented by sticks and marked according to the sequence.

T1 and T2 pore architectures form along the same threefold symmetry axis but on opposite sides of the protein cage (Figure 2A,B). In total there are four identical C3 symmetry axes in *Li*Dps, consequently one *Li*Dps dodecamer contains four T1 and four T2 pores. In T1 three symmetry-related monomers contribute primarily negatively charged residues (mostly glutamates) located on helix 3 and helix 4 (Figure 2A) while residues mainly located on helix 2 contribute to the formation of translocation pore T2. This second translocation pore was proposed by our group after the identification of Co^2+^ ions in the pore center but is still speculative as the contribution to ion translocation is not yet determined using, e.g., point mutants (terms T2, Figure 2B) [19].

T1 has been reported to be the main iron translocation channel in *Li*Dps and related Dps proteins [33]. The T1 pore forms a concave shape with a diameter of ∼1.2 nm at the pore entrance and 0.3 nm at the pore exit. The length of this pore is ~17 Å. Negatively charged Glu118, Asp121 and Asp126 and their symmetry-related residues at the outer pore surface of T1 form the surface potential to attract metal ions toward the protein complex (Figure 2C). The inner part of the pore includes three negatively charged Asp130 residues that contribute significantly to ion translocation by a direct and short interaction with the ions. It is predicted that these residues have an additional filter function to block the passage of hydrated ions as well as the entry of small organic molecules (Figure 2D). According to our recent experiments, these three Asp130 residues change their conformation dynamically in response to metal ions, which might assist the entry of ions into the cavity. From the entrance point of the cavity ions are further translocated toward the ferroxidase center via a distance of 23 Å. There are additional binding sites which we consider to be intermediate positions paving the way toward the FOC. These residues are Glu55 and Glu59 which are only 18 Å away from the point of ion entry into the cavity (Figure 2D).

### 3.2. Yb^3+^ Distribution at LiDps after 30 min Soaking Resembles a Dynamic Structure

Changes relative to the apo protein structure are induced when soaking Dps crystals in Yb^3+^ for 30 min at 10 mM (structure termed Dps_Yb_30). The crystal structure shows only four Yb^3+^ ions at the outer surface of T1 (three positions termed P1 and one position in the center termed P2) and one Yb^3+^ ion inside the pore (Figure 2C). The distances are 7.5 Å between P1 and P2 and also between P2 and P3, respectively (see Figure 2B,C). In solution, Yb^3+^ is coordinated by nine water molecules but these waters can be replaced by direct protein−Yb^3+^ interactions. In the outermost P1 positions, Yb^3+^ is directly coordinated to Glu118 in Dsp_Yb_30 at a distance of 3 Å. Distances of 3–3.5 Å are considered to be direct interactions, while larger distances around 6 Å indicate the presence of bridging water molecules, e.g., between Yb^3+^ and Glu118. Figure 2E depicts the superimposed residues of Apo-Dps and Dps_Yb_30 at the attraction area around the T1 pore. There are conformational changes of side chains such as in Glu118 of Apo-Dps directed to the outside of the T1 pore, while in Dps_Yb_30 the residues change direction toward the center of the T1 pore for coordination of the Yb^3+^ ion. The second Yb^3+^ ion is at P2, about a 7.5 Å distance from P1 aligned with the C3 symmetry axis, and here Yb^3+^ is coordinated by three Asp121 residues at a distance of 5 Å (likely an indirect or water-bridged interaction) and water molecules (Figure 2D). Interestingly, these coordinating water molecules mediating interactions to amino acids are an unusual feature and rarely observed in protein structures. The third Yb^3+^ at position 3 (P3) in Dps_Yb_30 is 7.5 Å away from P2 and binds through six carboxylate oxygens donated by three Asp130 and three water molecules to yield the ninefold symmetric coordination sphere (direct interactions; Figure 2D,F). Figure 2F shows a side view comparing the residues at the T1 channel of Apo-Dps and Dps_Yb_30. Although three Asp130 residues in Apo-Dps are directed toward the cavity, the Asp130 residues in Dps_Yb_30 orient to the T1 channel to coordinate a Yb^3+^ ion in the channel interior. Due to the rather limited exposure of Yb to the Dps crystals and the partial occupation of Yb positions, the Dps_Yb_30 structure is considered to be captured in a dynamic state which we believe is undergoing dynamic fluctuations of ions into the cavity due to electrostatically favorable interactions.

### 3.3. Yb^3+^ Ion Distribution at T1 in a Static Dps Structure

**Figure 3:**
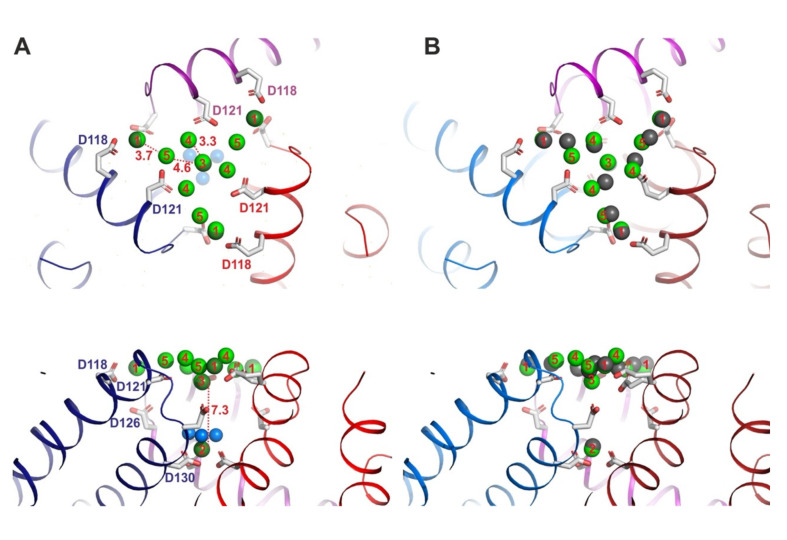
Architectures and uptake mechanism of Yb^3+^ ions through the T1 uptake pore of *Li*Dps with Yb^3+^ after 120 min of soaking (Dps_Yb_120). (**A**) Architecture of the T1 pore viewed from the top of Dps_Yb_120 in cartoon representation (upper figure). Yb^3+^ ions are shown as light green spheres with positions termed 1–5. The residues of one subunit interacting with ions along the 3-fold axis are represented by sticks and marked according to the amino acid sequence. Architecture of the T1 pore viewed from the side (lower figure). (**B**) Architecture of the T1 pore viewed from the top with superimposed Yb^3+^ and La^3+^ ions. La^3+^ ions and water molecules are shown as black and blue spheres. The dotted red line indicates the distance from position 2 to position 3. Architecture of the T1 pore viewed from the side (lower figure).

After soaking Dps crystal for 120 min in Yb^3+^ at 10 mM (called Dps_Yb_120 derivative), a total of 11 Yb^3+^ ions at the T1 pore were confirmed by anomalous and difference electron densities (Figure 3A). Although the entrance area that is formed by Glu118, Asp121 and Asp126 have only four Yb^3+^ ions on Dps_Yb_30, there are additional Yb^3+^ ions at positions P4 and P5 nearby Asp121 (distance: 4.5 Å to position 4 and 3.0 Å) on Dps_Yb_120 (Figure 3). The residues of Glu118 on Dps_Yb_120 are also directed to the C3 axis on the T1 pore to directly coordinate and stabilize Yb^3+^. On the contrary, water molecules are not modelled due to the crowded and undefined electron density at P4 and P5. The distances of P4 and P5 from Asp121 would indicate that the Yb^3+^ ions at positions 4 and 5 could be coordinated through water molecules. It is difficult to stabilize Yb^3+^ ions at positions 4 and 5 without a water molecule bridge (coordination) with Asp121 due to the larger distance between the side chain and metal ion. The comparison image (Figure 3A) of Dps_Yb_120 and Dps soaked with La^3+^ shows there are La^3+^ ions in similar positions to P4 and P5. The data indicates that the La^3+^ ions at these similar positions have water molecules, which supports the idea that Yb^3+^ ions are coordinated by water molecules. The other difference between Dps_Yb_120 and Dps soaked with La^3+^ is the existence of Yb^3+^ ions along the C3 axis. There are no La^3+^ ions along the C3 axis except the La^3+^ ion at P3. On Dps_Yb_120, there is an additional Yb^3+^ ion at P3 (Figure 3B), which is the same position in Dps_Yb_30. Along the C3 axis, the Yb^3+^ ion at P3 is always coordinated with six carboxylate residues from three Asp130 and three water molecules on Dps_Yb_30, Dps_Yb_120 and Dps soaked with La^3+^. The direction of three Asp130 side chains are oriented to the center of the T1 pore, which is the same conformation as found in Dps_Yb_30 and Dps soaked with La^3+^, but not in Apo-Dps.

### 3.4. Yb^3+^ May Use T2 Channels and Reach FOC via Intermediate Sites

**Figure 4:**
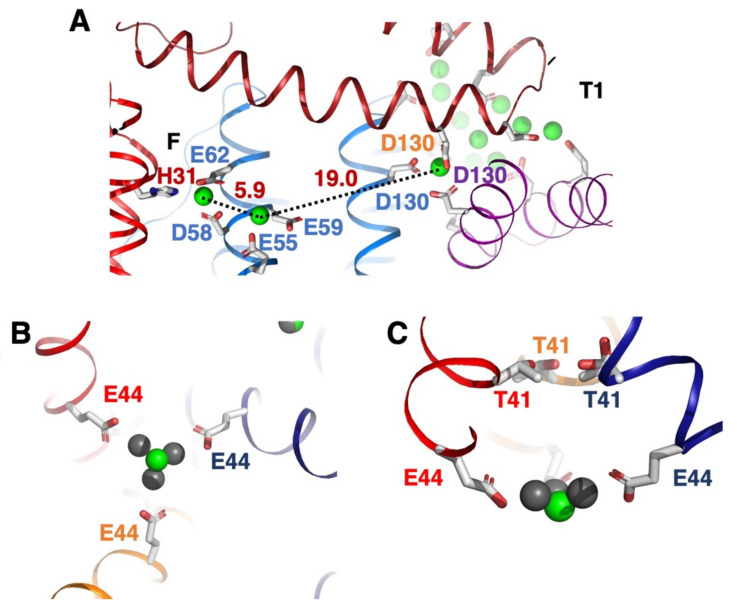
(**A**) Architecture of T1 to FOC of Dps_Yb_120 in cartoon representation. Yb^3+^ ion is shown as a light green sphere. The residues of one subunit along the 3-fold axis are represented by sticks and marked according to the amino acid sequence. (**B**) Architecture of the T2 pore viewed from the inside of Dps_Yb_120 in cartoon representation. (**C**) Architecture of the T2 pore viewed from the side.

After Yb^3+^ ions have reached the cavity from the T1 pore, Yb^3+^ ions are distributed to the FOCs. Although there are four T1 pores and a total of 12 FOC sites in one Dps protein, there are 3 FOCs nearest to one of the T1 pores (Figure 4A). Yb^3+^ ions from T1 can be attracted to the nearest FOC by electrostatic interactions and be coordinated with Glu55, Asp58, Glu59 and Glu62 from one monomer, and His31 from the neighboring subunit to form the FOC (Figure 4B). In the Dps_Yb_ structures we also identified an intermediate site of two glutamate residues Glu55 and Glu59 at a distance of 5.9 Å from the FOC ion position. FOCs in Dps proteins can bind a variety of different ions such as Ni, Zn, Co, Fe but also La and Yb [19]. There is one more Yb^3+^ position at T2 in Dps_Yb_30 and Dps_Yb_120 (Figure 4A,B). In Dps, there is a T2 pore. In the T2 pore on Dps_Yb_30 and Dps_Yb_120, there is no Yb^3+^ along the C3 symmetry axis except in the inner part of the pore (Figure 4C), although there are two Co^2+^ ions at the T2 pore in the case of Co^2+^. Interestingly, there is only one Yb^3+^ ion that binds to three Glu44 in Dsp_Yb_30 and Dsp_Yb_120. In the case of Dps soaked with La^3+^, the La^3+^ ion is bound to the three shared positions as indicated by the occupancy data (Figure 4C) [19].

## 4. Discussion

The aim of our research is to investigate the dynamic and static states of ion translocation in the Dps protein from *L. innocua*. Dynamic states characterize proteins with respect to changes between the apo and the static state. While the apo state is essentially an ion-free protein cage, the static state reflects the protein complex entirely filled with ions and further ion flux is suppressed due to electrostatics of crowding. Biomineralization in Dps proteins is time dependent but also a multistep process starting with ion attraction, by negatively charged residues, toward the surface of the protein. This is followed by translocation through the pores, oxidation at the FOCs, and nucleation at nucleation centers leading to biomineralization inside the cavity. In X-ray diffraction data, moving Yb^3+^ ions cannot be observed but snapshots of the ion states can be taken after freezing the crystals. In this study we aimed to identify this process in a time-dependent manner using crystals soaked with Yb^3+^ for 30 and 120 min. Initially our structures show the attraction of Yb^3+^ toward the outer surface where two stable interactions are observed in the dynamic state, with additional interactions occurring after longer incubation. However, Yb^3+^ ions are expected to be attracted to the T1 entrance area by charge attraction without stable states in Dps_Yb_30. In Dps_Yb_30 some Yb^3+^ sites at P4 and P5 are not stabilized by channel amino acids, yet some of the Yb^3+^ ions are attracted by the negative charge gradient from the inner cavity of Dps [33]. By contrast, after 120 min, Yb^3+^ ions would be stabilized in T1 pores. Therefore, we can observe six additional Yb^3+^ ions at positions P4 and P5. Additional Yb^3+^ ions were positioned without direct coordination with amino acid residues and due to their proximity, it is likely that they may be present with low occupancies. Inside the channel, e.g., in *Microbacterium arborescens* Dps, iron ions coordinated with six water molecules were observed, and these ions did not show a direct interaction with the channel wall residues, similar to the situation of Co and Zn in *Li*Dps for P2 [34]. When iron is coordinated by water molecules the interaction with the charged protein surface is reduced and dynamic processes such as translocation through pores might be easier.

In spite of the stability of additional Yb^3+^ ions at P4 and P5 after 120 min, in the corresponding Dsp_Yb_30 and Dsp_Yb_120 data sets one Yb^3+^ or La^3+^ ion is found at P1 of the T1 pore via a direct interaction to Glu118 and an indirect interaction to Asp126. Binding of Yb at Glu118 introduces a conformational change in Glu118. In the La^3+^ structure, the direct coordination has been confirmed, which means the coordination properties of metal ions affect the conformation change of amino acids. This kind of trend, relevant to coordination properties with amino acids, can be explained by the other parts in Dps. P2 is only stabilized by indirect interactions to three residues of Glu121 (Figure 2C,D).

In Apo-Dps, three Asp130 residues forming the channel/cavity transition are directed toward the cavity of Dps [19]. In the Dsp_Yb_30 and Dsp_Yb_120 structure one Yb^3+^ is connected to three Asp130 residues with the side chains directed toward the pore center. Here, Yb^3+^ shows a ninefold coordination sphere using six oxo atoms of three Asp130 residues and three water molecules to fill this electrostatic environment. The Yb^3+^−Asp130 interaction is obviously not static but dynamic and Asp130 releases Yb^3+^ into the cavity of Dps through conformational changes. The coordination properties and the amino acid dynamics maintain this specific feature on the T1 pore to generate metal ion flows. In contrast to Yb^3+^ and La^3+^, both of which are coordinated by Asp130 inside the pore, divalent ions such as Co^2+^ or Zn^2+^ induce a conformation whereby the residues are facing the cavity with both ions being captured inside the cavity [19]. In unpublished data, using the dynamic state of an iron-enriched structure, an asymmetric arrangement of three Asp130 residues with two side chains pointing inside the cavity while one was oriented toward the channel is another logical intermediate which enables the rapid exchange of ions, thus demonstrating the plasticity of this side chain in ion exchange.

**Figure 5:**
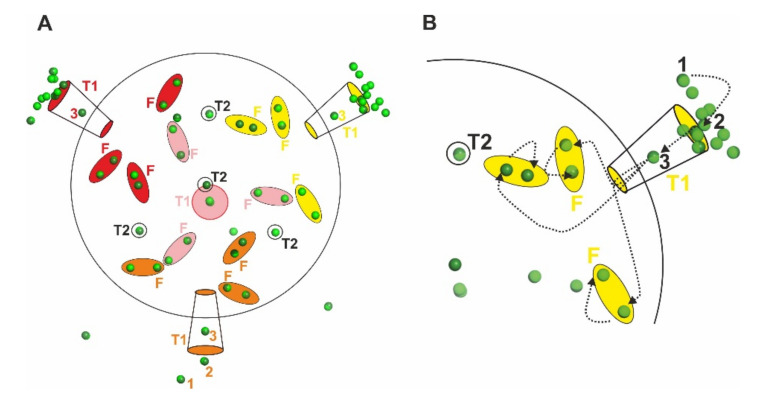
(**A**) Schematic representation of Yb^3+^ ions localized at T1 pores, T2 pores and FOCs in Dps_Yb_30 (dark green spheres) and Dps_Yb_120 (light green spheres). A big black circle indicates the outline of the Dps protein. Each cylinder-shaped and encircled color group (red, pink, yellow and orange) shows one set of one T1 pore (written as T1) and three FOC sites (written as F). There are four T1 pores per Dps protein. The number of Yb^3+^ ions shown here (dark green for Dps_Yb_30 and light green for Dps_Yb_120) show a difference on four of the T1 pores in this schematic. Two of four T1 pores are partially closed by interactions with neighboring Dps proteins in the crystallographic sphere, which restricts Yb^3+^ ion access toward the T1 pores in pink and orange colors. There is one Yb^3+^ on each T2 pore as shown by four black circles (written as T2) in the Dps protein. (**B**) One T1 pore spreads Yb^3+^ ions over three FOC sites. Dotted arrows indicate ideal ways to transport Yb^3+^ ions from T1 to the FOCs.

Upon further inspection of the structures, we also noticed additional sites in between the Asp130 residue and the FOC. There are Glu55 and Glu59 residues nearby the FOC, which attract Yb^3+^ by their negative charges (Figure 5). These residues may help to attract ions toward the FOC, but also to cross the distance between the Asp130 residue and the FOC in a more effective way. From this site the ions may cross the remaining distance of about 6 Å to the FOC.

Probably the only conserved position in all Dps proteins, attracting and binding all metal ions independent of their coordination sphere, is the FOC center (Figure 5). The FOC center can stabilize metal ions through coordination of His31, Asp58 and Glu62. However, these residues can also strongly adapt to the properties of the metals, e.g., glutamates and aspartates can provide either one or two oxo atoms for coordination. Co^2+^ ions require a coordination sphere of six and, indeed, in the crystal structure the Asp58 provides two oxo atoms for binding, Glu62 one and His31 also one, with two additional water molecules completing the sphere. For the Yb^3+^ ion at the FOC center, Asp58 and Glu62 provide two oxo atoms, His31 another, and four waters complete the coordination sphere. This observation not only shows the plasticity of the FOC but also demonstrates that completing the coordination sphere of a metal strongly guides those ions to one site in preference to other sites which cannot offer the required coordination sphere.

Similarly, the Yb^3+^ position with three Glu44 residues at the inner side of the T2 pore places only one Yb^3+^ ion through the negatively charged amino acids. In the Dps soaked with La^3+^ structure there are three La^3+^ ions at almost the same position around the Yb^3+^ position at the T2 pore, however, the occupancy is 0.3 for each La^3+^, while the occupancy can reach 1 for Yb ^3+^. The difference between Yb^3+^ and La^3+^ might be due to the difference of the coordination ability between Yb^3+^ and La^3+^. The position of Yb^3+^ from Glu44 is 4.5 Å (greater than 2.7 Å, suggesting that there are water molecules around Yb^3+^ between the position of Yb^3+^ and Glu44). The coordination properties and diameter of the metal ions would affect the binding and unbinding of the metal ions with amino acids.

On the Dps interfacial surface, the binding of metal ions with amino acids is completely in accordance with the coordination properties of the metal ions and the amino acids. Electrical charges of the metal ions and amino acids can generate interactions between them, and therefore, Yb^3+^ and Co^2+^ are attracted to the T1 channels as found in the dynamic state of Yb^3+^ after 30 min. After 120 min, additional Yb^3+^ ions would be stabilized by the amino acids on Dps, and Yb^3+^ ions stabilized after 30 min can stabilize additional Yb^3+^ ions. Data from the Yb^3+^ and La^3+^ ions showed similar binding properties on T1 and FOC, but the difference in coordination diameter can be demonstrated by the difference in the number of Yb^3+^ and La^3+^ ions on the T2 sites.

## 5. Conclusions

In this report, we demonstrate the structures of *Li*Dps after soaking with Yb^3+^ for 30 or 120 min. Depending on the soaking time, the distribution of Yb^3+^ ions shows differences mainly around the T1 pore. This result suggests that the T1 pore attracts Yb^3+^ ions after 30 min, however, Yb^3+^ ions would dynamically move through the T1 pore, and their distribution remains dynamic until the cavity is filled. After soaking the crystals for a long time, the Yb^3+^ ions will reach a stable or static state which does not allow further changes. The coordination properties of Yb^3+^ ions and amino acids affects specific binding to the interfacial region of *Li*Dps at FOC sites and at the exit site of T2 pores. Our observations may assist in the design of surfaces for inorganic nanoparticle formation with tailored (e.g., kinetically optimized) properties to produce magnetic and luminescence particles for medical and nanotechnological applications.

## Data Availability

Data are available in a publicly accessible repository.

## Supplementary Materials

The following is available online at https://www.mdpi.com/article/10.3390/biomedicines9080914/s1, Table S1: Data collection and refinement statics.
