# Supplementary material for: Time-Resolved Studies of Ytterbium Distribution at Interfacial Surfaces of Ferritin-like Dps Protein Demonstrate Metal Uptake and Storage Pathways
Source: Biomedicines. 2021 Jul 29;9(8):914. doi: 10.3390/biomedicines9080914 (PMC8389677; doi:10.3390/biomedicines9080914)
Supplement: Supplementary file 1 [file biomedicines-09-00914-s001.zip › biomedicines-1257533-supplementary.pdf]

Supplementary Material

Table S1

Data collection and refinement statistics

	$Dps_{Yb}30$	$Dps_{Yb}120$
**Data collection**		
Space group	$P4_12_12$	$P4_12_12$
Cell dimensions		
a, *b*, *c* (Å)	86.99, 86.99, 275.01	87.12, 87.12, 275.26
α, β, γ (°)	90, 90, 90	90, 90, 90
Resolution (Å)	50-1.8 (1.9-1.8)	32-1.8 (1.9-1.8)
R_{sym} or R_{merge}	0.18 (2.5)	0.13 (2.2)
CC* in outermost shell	12.1	18.4
$I / \sigma I$	9.49 (0.5)	9.3 (0.5)
Completeness (%)	95.5 (76.9)	95.3 (76.2)
Redundancy	8.6 (7.2)	7.2 (5.7)
**Refinement**		
Program	**PHENIX**	**PHENIX**
Resolution (Å)	50-1.8 (1.81-1.80)	32-1.8 (1.81-1.80)
No. reflections	178954	178952
R_{work} / R_{free}	0.17/0.21	0.19/0.21
No. atoms (all)	8113	7768
Protein	7322	7374
Water	688	394
Metal	27	32
B-factors		
Protein	41.7	41.1
Water	46.9	45.1
Metal	99.6	107.8
R.m.s. deviations		
Bond lengths (Å)	0.013	0.003
Bond angles (°)	1.3	0.52
**Ramachandran statistics**		
Residues in favored region No (%)	98.6	98.7
Residues in allowed region No (%)	1.33	1.3
Residues in outlier region No (%)	0	0

**PDB-entry**	

*Values in parentheses are for highest-resolution shell.
